# Promoter Targeting RNAs: Unexpected Contributors to the Control of HIV-1 Transcription

**DOI:** 10.1038/mtna.2014.67

**Published:** 2015-01-27

**Authors:** Kazuo Suzuki, Chantelle Ahlenstiel, Katherine Marks, Anthony D Kelleher

**Affiliations:** 1 Immunovirology laboratory, St Vincent's Centre for Applied Medical Research, Darlinghurst, Australia; 2 The Kirby Institute, University of New South Wales, Sydney, Australia

## Abstract

In spite of prolonged and intensive treatment with combined antiretroviral therapy (cART), which efficiently suppresses plasma viremia, the integrated provirus of HIV-1 persists in resting memory CD4^+^ T cells as latent infection. Treatment with cART does not substantially reduce the burden of latent infection. Once cART is ceased, HIV-1 replication recrudesces from these reservoirs in the overwhelming majority of patients. There is increasing evidence supporting a role for noncoding RNAs (ncRNA), including microRNAs (miRNAs), antisense (as)RNAs, and short interfering (si)RNA in the regulation of HIV-1 transcription. This appears to be mediated by interaction with the HIV-1 promoter region. Viral miRNAs have the potential to act as positive or negative regulators of HIV transcription. Moreover, inhibition of virally encoded long-asRNA can induce positive transcriptional regulation, while antisense strands of siRNA targeting the NF-κB region suppress viral transcription. An in-depth understanding of the interaction between ncRNAs and the HIV-1 U3 promoter region may lead to new approaches for the control of HIV reservoirs. This review focuses on promoter associated ncRNAs, with particular emphasis on their role in determining whether HIV-1 establishes active or latent infection.

## Introduction

It is well known that noncoding RNAs (ncRNAs) are implicated in a wide variety of cellular processes through posttranscriptional regulation of protein expression. In addition, there is increasing evidence pointing to their role in transcriptional gene regulation. High-throughput sequencing technology which allows analysis of the global transcriptome has revealed that over 90% of genomic DNA is utilized for transcription.^1,2,3,4^ Out of the total transcriptome, only a small portion (~2%) is translated into proteins. Therefore, ncRNAs represent a large portion of the transcriptome in mammals,^2^ and there is growing evidence that these transcripts function to regulate gene expression, especially in developmental pathways and in response to environmental stressors, such as viral infection.^5^ However, the expression levels of these ncRNAs are extremely low compared to mRNAs, perhaps consistent with their involvement in regulatory processes.^2^ NcRNAs can be categorized as infrastructural and regulatory. Ribosomal and transfer RNAs belong to infrastructural ncRNAs, while regulatory ncRNAs are broadly divided into two classes: small ncRNAs (< 200 nucleotides) and long ncRNAs (>200 nucleotides).^6^ The small ncRNAs are further classified into several subcategories: microRNA (miRNAs), 19–24 nucleotides in length, short interfering RNAs (siRNAs, ~22 nucleotides), and antisense RNAs (asRNAs, <200 nucleotides). The long noncoding RNA (lncRNA) category includes intergenic ncRNA, pseudogene transcripts, and long antisense RNAs (long asRNA, >200 nucleotides), which are also known as long antisense noncoding RNAs (antisense lncRNAs)^2,4,6,7,8,9^

Transcription of the integrated form of HIV-1 is controlled by a range of epigenetic mechanisms. Several recent studies have found that ncRNAs play a role in this process. After HIV-1 integration, the extremely strong HIV-1 promoter drives active transcription of viral mRNA in the majority of CD4^+^ T cells. In these cells, the viral transactivator protein, Tat, interacts with a hairpin structure in the viral RNA called the Transactivation response element (TAR), resulting in more efficient recruitment of RNA polymerase to drive strong viral transcription.^10,11^ However, in a portion of resting memory CD4^+^ T cells latent infection is established. In these cells, HIV-1 transcription is shut down, even in the presence of a competent integrated HIV-1 genome. Latent infection is associated with the formation of closed chromatin or heterochromatin, characterized by deacetylation followed by methylation of specific residues in the histone 3 molecules (*e.g.*, di-methylation of the lysine 9 of histone 3; H3K9me2) associated with the HIV-1 promoter region. These processes are mediated by specific deacetylases and methyltransferases recruited to the chromatin. In contrast, the reactivation of latent HIV-1 is associated with the formation of euchromatin (also referred to as open/lytic chromatin) in this region, characterized by acetylation of histone 3 tails (H3Ac).^12,13^

The precise mechanisms underlying establishment of latent infection are largely unclear. Following reverse transcription, HIV-1 DNA integrates into the human genome and the proviral DNA is organized within host chromatin, which forms three nucleosomes at precise locations in the HIV-1 5′ LTR. The location of nucleosomes (nuc-0, nuc-1, and nuc-2) in the HIV-1 promoter is reproducible and predictable.^14,15^ The formation of heterochromatin in the HIV-1 5′ LTR in latently infected cells is believed to be regulated by host factors, particularly a lack of T-cell activation and a relative paucity of host transcription factors that help drive HIV-1 transcription.^16^ Recent research reveals that promoter-associated ncRNA, including miRNAs, short and long asRNAs, as well as siRNAs, contribute significantly to the regulation of heterochromatin formation associated with the HIV-1 promoter.^17,18,19,20^ This review will focus on promoter-associated ncRNAs that affect HIV-1 gene regulation, specifically in terms of controlling HIV-1 transcription, with particular emphasis on regulation of HIV-1 latent infection, whereby both negative and positive regulation can be induced by viral promoter-associated ncRNAs.

## Current limitations of HIV-1 therapy

Despite combined antiretroviral therapy (cART) bringing a revolution in the treatment of HIV-1 infection, which has turned a universally fatal illness into a chronic condition, it has some limitations. CART potentially prevents further rounds of viral replication, rapidly suppressing plasma viral load (pVL) to levels where only ultrasensitive assays are capable of detecting low levels of HIV RNA in plasma,^21^ whilst concurrently allowing immune reconstitution.^22^ However, the integrated HIV-1 genome is not directly affected by cART. The integrated HIV-1 provirus persists for the life span of the infected cell and therefore, long-lived cells act as a reservoir from which viral replication can arise when cART is interrupted.^23,24^ It is believed that much of this reservoir is in a latent, transcriptionally silenced form. The best characterized part of the reservoir is found within resting memory CD4^+^ T cells.^21,25,26,27,28^ Viral replication, as demonstrated by increases in pVL, resumes rapidly upon cessation of cART in most cases. Interrupting therapy is associated with a significantly worse prognosis and so once commenced, life-long therapy is required. This, in turn, is associated with various limitations including: a variety of cumulative side effects and toxicities, such as dysregulation of lipid metabolism, increased prevalence of cardiovascular disease and reduction in bone density. Furthermore, suboptimal compliance with prolonged therapy fosters the development of resistance mutations.^29,30,31^ Another limitation is cost and sustainability. As individuals live longer on cART, the cost per person increases and in addition, since the number of individuals on therapy increases, the cost per population also increases. These combine to place substantial pressures on health care systems and budgets.^22^

**Figure 1:**
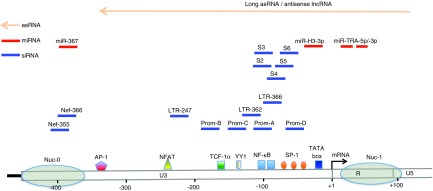
**Schematic of noncoding RNAs interacting with the HIV-1 promoter region. After HIV-1 is integrated into the human genome, nucleosomes are precisely embedded within the U3 promoter region of 5′ LTR. Nuc-0 is located at the start of the U3 promoter region and Nuc-1 is located at the start of the “R” region.** Transcription is initiated in the R region, indicated by the arrow.^121,122^ Various host transcriptional regulators interact with the U3 promoter region; predicted binding sites for transcription activators such as Nuclear Factor-κB (NF-κB), Nuclear Factor of Activated T-cell protein (NFAT), Specificity Protein 1 (SP1), TATA-box, T-cell–specific factor-1α (TCF-1α), and Activator Protein 1 (AP-1) are shown. The binding site of transcription repressor, Yin Yang 1 (YY1), is also shown.^121^ Various species of promoter associated ncRNAs including, long antisense RNAs (long asRNA)/antisense long noncoding RNAs (antisense lncRNAs) (light orange), miRNAs (red), and siRNAs (blue) are shown above the U3 promoter region (see text for detail).

These limitations suggest that alternative approaches are required to combat HIV-1 infection, particularly with regard to mechanisms that reduce the latent reservoir. However, there is still a relatively poor understanding of the mechanisms underlying the induction and maintenance of latency. Furthermore, the precise molecular mechanisms operating *in vivo* that drive reactivation from latent infection are largely unclear.^10^ During active replication, HIV-1 transcription is driven by a number of host and viral factors. The HIV-1 5′ LTR contains various motifs for a range of host transcription factors, including NF-κB, NFAT, and Sp-1 (**Figure 1**). The major viral activator of viral transcription is the viral transactivation protein, Tat, which massively upregulates the efficiency of viral transcription by binding to TAR, a hairpin structure within the RNA transcript of the 5′ LTR.

In latently infected cells, HIV-1 transcription is inhibited, blocked, or restricted to subgenomic transcripts. Several hypotheses related to epigenetic mechanisms have been suggested to explain this postintegration latent phase of viral infection. These include (i) chromatin in the HIV-1 promoter is epigenetically modified to a compacted-closed state reducing the ability of host transcription factors to bind the promoter, thereby inhibiting recruitment of RNA-polymerase II, which results in inefficient promoter driven transcription; (ii) a lack of critical host transcription factors due to the lack of cellular activation in resting memory CD4^+^ T cells; (iii) suboptimal production or lack of posttranslational modification of Tat, resulting in inhibition of efficient transcription of full length viral RNA; and (iv) the location of the integrated HIV-1 genome, whereby HIV-1 is integrated into a transcriptionally silenced region of the host genome and viral transcription is therefore also repressed.

Recently, it has been suggested that rather than viral suppression, the ultimate goal of therapeutic approaches should be to achieve a “functional cure.” This is a clinical state where pVL is maintained below detection levels in the absence of cART. Currently, the most studied approach is the so-called “Kick and Kill Approach.” The object is to reactivate the latent virus in the presence of cART. It was hoped that viral reactivation itself would either directly kill the infected cell or that expression of viral proteins would make the cell a lethal target of the immune system, while the presence of cART would prevent infection of new cells. This would eventually result in reduction in the size of the reservoir or its complete depletion. A number of clinical trials exploring “Kick and Kill” interventions have been conducted in patients on cART with the endpoints being the extent of viral reactivation and the resulting reduction in viral reservoir.^32,33^ The interventions explored so far have included pan-T-cell activation through the use of OKT3,^34^ recombinant IL-2,^10^ or IL-7.^35,36^ Other candidates include Bryostatin, which acts through activation of the protein kinase C (PKC) pathway and has so far only been explored *ex vivo*.^37^ In each case, their mode of action is characterized by the activation of CD4^+^ T cells in general. The underlying intention is to increase the expression of host transcription factors that drive activation of the virus. Each of these approaches has had very limited success in reducing the reservoir.

More recently, a different approach, aimed at activating the virus by altering the epigenetic profile of the latent virus, has employed Histone deacetylase (HDAC) inhibitors (HDACi), such as valproic acid (VPA), suberoylanilide hydroxamic acid (SAHA, vorinostat), romidepsin, and parabinostat.^38,39,40,41,42,43^ Although these drugs have the ability to reactivate some integrated virus *in vitro*, so far, these agents have resulted in quite limited viral reactivation *in vivo*, while causing significant activation of a range of host genes.^13,44^ This suboptimal response suggests that a more focused approach is required to increase the specificity of such interventions. This is supported by the recently reported observation that vorinostat can inhibit cytotoxic T-lymphocyte (CTL) function.^45^ This interaction has the potential to substantially limit the effectiveness of HDACi in the “Kick and Kill Approach,” as it was hoped that CTL would mediate the “killing” once latently infected cells were “kicked” into expressing viral antigens.^45^

Taken together, these results suggest that further understanding of the mechanisms driving HIV-1 latency and the precise molecular mechanisms required to specifically activate HIV from latent infection are required for the rational design of interventions that impact on the latent reservoir. Recent reports have suggested that ncRNAs including miRNA, siRNA, and asRNA, are associated with the regulation of the viral latency.^46,47^ Therefore, these molecules may provide the actual tools, or at least insights into mechanisms, that will allow specific regulation of viral latency.

## Transcriptional activation and suppression through promoter associated miRNAs

More than a decade ago, miRNAs were reported to play a key regulatory role in the inhibition of translation of mRNA into protein.^48^ Dicer, an RNase III enzyme, processes immature miRNA stem-loop structures into mature miRNAs ~22 nucleotides long.^49^ The mature miRNAs are loaded into an Argonaute (Ago) protein forming an RNA-induced silencing complex (RISC). The seed region (nucleotides 2–8) of a miRNA interacts with the 3′-UTR of its complementary target in a mRNA transcript. This is the key determinant in translational repression. Because the length of the seed region is short (~7 nucleotides long), one miRNA is capable of regulating multiple mRNAs and their subsequent translation into protein.^50,51^

A second function has been attributed to miRNA. In this case, miRNA act as a posttranscriptional repressor, cleaving the target mRNA. In this case, there is a requirement for near perfect homology between the miRNA and sequences within the mRNA, as has been reported in the determinants of function for short interfering (si)RNA^52^ (see below).

Translational regulation and cleavage of mRNA mediated by miRNA are forms of posttranscriptional regulation. Both occur in the cytoplasm. Recently, miRNA has also been reported to act as a transcriptional regulator. This function occurs in the nucleus. In this case, the miRNA interacts directly with the gene promoter. For instance, miRNA-320 (miR-320) induces transcriptional gene silencing (TGS) of the gene polymerase (RNA) III (DNA directed) polypeptide (*POLR3D*) by interacting with its promoter region.^52^ This was demonstrated in three separate cell lines: bEnd.3 cortex cells, HeLa human cervical carcinoma cells, and HEK-293 human embryonic kidney fibroblast cells. The effect appeared dependent upon miR-320 binding to Argonaute-1 (Ago1). This complex resulted in elevated levels of Polycomb group component Enhancer of Zeste-2 (EZH2) and histone H3 lysine 27 tri-methylation (H3K27me3) at the targeted promoter.^52^ Similarly, TGS of the progesterone receptor gene can be mediated by miR-423-5p,^53^ however, the mechanism by which it induced TGS appears to be somewhat different.^52^ In this case, miR-423-5p was shown to be loaded onto Argonaute-2 (Ago2), rather than Ago1. This was associated with enrichment of H3 lysine 9 di-methylation (H3K9me2) in chromatin associated with the target promoter.

While these are novel observations demonstrating transcriptional repression by miRNAs, other observations suggest that certain miRNAs are capable of transcriptional activation, thereby facilitating transcription rather than inhibiting it. The first discovery of transcriptional activation was reported in PC-3 cells, a tumor cell line. Expression levels of both E-cadherin and cold-shock domain-containing protein C2 (*CSDC2*) were increased through the action of promoter associated-miRNA-373 (miR-373).^54^ Transcriptional activation was associated with enrichment of RNA polymerase II in the promoter regions of both genes.^54^ Subsequently, transcriptional activation by promoter associated miRNAs has been reported in studies of three prostate cancer cell lines; LNCaP, PC-3, and Du145. The authors demonstrated that miRNA-205 (miR-205) induced the expression of interleukin (IL) tumor suppressor genes *IL24* and *IL32* by targeting specific sites in their promoters, which resulted in increases in both the messenger RNA and protein levels. The induction of *in vitro* transcription by miR-205 was linked with enrichment of histone markers for transcriptionally active promoters in the *IL24* and *IL32* genes.^55^

## Interaction between host miRNAs and HIV-1 transcription

Recent studies on the role of miRNAs in HIV-1 pathogenesis suggested that five host derived miRNAs (miR-28, miR-125b, miR-150, miR-223, and miR-382) were enriched in resting primary CD4^+^ T cells compared with activated CD4^+^ T cells, and that this group of miRNAs played a role in establishing viral latency.^56^ These findings have become controversial. This is primarily due to only partial concordance of several studies attempting to reproduce these findings. One study confirmed high levels of expression of all five miRNAs in freshly isolated monocytes.^57^ In another intensive *in vitro* study of monocyte derived macrophages (MDMs), four miRNA (miR-28, miR-150, miR-223, and miR-382) out of these five “anti-HIV-1 miRNAs” were suggested to have the potential for antiviral function, since expression levels of these miRNAs were elevated in MDMs cultured in the presence of type 1 interferon IFN-α or IFN-β, which resulted in the suppression of HIV-1 replication.^58^ *In vitro* treatment of lymphocytes with that cocaine has been associated with downregulation of miR-125b,^59^ while heroin was associated with downregulation of all five “anti-HIV-1 miRNAs.”^60^ Both treatments enhanced HIV replication. Our group has also reported that the expression of two of these miRNAs (miR-150 and miR28-5p) is elevated in freshly isolated CD4^+^ T cells from healthy controls, when compared to HIV-1 infected patients.^61^ However, in direct contrast several studies could not reproduce the high level expression of any of the five “anti-HIV-1 miRNAs” in CD4^+^ T cells.^59,62,63,64^

There is also debate regarding the extent of correlation between the detection of these miRNAs and their actual *in vivo* effects. Interestingly, it has been demonstrated that despite possible binding sites for the seed sequences of these “anti-HIV-1 miRNAs” in the Nef-3′ LTR consensus sequence, direct targeting of the HIV 3′-UTR only showed a modest effect on HIV-1 replication.^62,65,66^ In our opinion, the five identified miRNAs show some degree of suppression, warranting further study to definitively characterize the extent of this effect.

Other contributions to the interplay between host miRNA and HIV pathogenesis include the effect of these molecules on the HIV Tat protein and cellular cytokines. Recently, studies have identified an impact of cellular miRNAs on Tat function. MiR-34a, miR-182, and miR-217 can modulate Tat-induced HIV-1 long terminal repeat (LTR) transactivation by targeting host gene transcripts, rather than through direct interaction with the HIV-1 genome,^67,68,69^ MiR-34a can modulate in Tat induced LTR transactivation through the host factor of SIRT1/NF-κB pathway.^67^ MiR-217 has also been shown to modulate Tat induced LTR transactivation by downregulation of the host protein SIRT1^68^ and MiR-182 modulates Tat induced LTR transactivation via downregulation of host factor NAMPT.^69^ Several studies have also reported that host cellular miRNAs can reduce expression of cellular factors, which play a role in HIV-1 replication.^62,67,68,69,70,71^ MiR-17/92 has been shown to suppress HIV-1 transcription through reduced expression of the histone acetyltransferase Tat cofactor, PCAF.^72^

It has also become apparent, especially in an *in vivo* environment, that cytokine expression can be altered by modulation of host miRNAs in HIV-infected cells.^65,66,73^ IL-10 expression is elevated in CD4^+^ T cells after HIV-1 infection *in vitro* and is associated with progressive disease *in vivo*. It is noteworthy that the expression levels of several let-7 miRNAs are significantly decreased in these HIV-1 infected cells. Decreased let-7 miRNAs could be one possible explanation for increased levels of IL-10 in patients with progressive disease. Further, the expression of the transcriptional repressor Blimp1 is also elevated in progressive HIV-1 infection compared to nonprogressive disease, and miR-9 levels are significantly decreased. MiR-9 targets PRDM1 (the mRNA precursor to Blimp-1 protein), therefore decreased levels of miR-9 expression could allow increased Blimp-1 expression, and the resultant reduced IL-2 and increased IL-10 expression in CD4+ T cells, all which are observed in progressive HIV-1 infection.^65,66,73^ Furthermore, toll-like receptors (TLR) 7 and 8, which can recognize cellular miRNAs, may contribute to chronic immune activation in HIV-1 infected patients.^65^ Several studies have revealed that stimulation of TLR-3 by cellular miRNAs (miR-28, -125b, -150, -223, -382, and -155) in macrophages resulted in elevated secretion of type 1 IFN, which can augment HIV-1 replication *in vitro*.^58,74,75^ Clearly, the interplay between host cellular miRNAs and HIV-1 pathogenesis occurs through a complicated combination of multiple pathways and requires further investigation to fully understand their role in modulating infection and control of HIV-1 infection.^73,76,77,78,79,80,81,82,83,84^

## Interaction between viral miRNAs and HIV-1 transcription

Certain viral derived miRNA have also been reported to act as direct transcriptional regulators^19,85,86,87,88,89^ (**Figure 1**). Initial reports suggested that a miRNA derived from the *nef* coding region, miR-N367, acted in the U3 region of the 5′ LTR to suppress viral transcription in human T cells.^86^ Recently, the production of viral miRNAs, processed by Dicer, from the transactivation responsive (TAR) stem loop structure have been reported by several groups^87,88,89^ (**Figure 1**). These data, derived using 293 cells following transfection of a pLTR-luc construct, suggested that a TAR-derived miRNA is capable of regulating viral gene expression at the transcriptional level and is associated with alterations in the epigenetic profile.^87^ Another study revealed that each strand of the stem loop of TAR region is able to produce miRNAs, miR-TAR-5p, and miR-TAR-3p, and the silencing ability of miR-TAR-3p is superior to that of miR-TAR-5p, as measured by suppression of luciferase activity driven by LTR promoter.^88^ These results were also supported by suppression of viral production from CD4^+^ T-cell lines.^88^ TAR-derived miRNA may also interact with host genes. One study has shown that TAR miRNA protects infected cells from apoptosis by downregulating proapoptotic cellular genes, ERCC1 and IER, extending their life span and thereby allowing increased viral replication.^89^ Currently, the detection of virally derived miRNAs is controversial. While certain deep sequencing technologies were able to detect very low level production miRNAs derived from *nef* gene and TAR-stem loop regions,^19,90^ studies using different detection methods have failed to detect these miRNAs.^91,92,93^ One extensive study which used very sensitive deep sequencing technology showed that HIV-1 may not express any biologically functional viral-derived miRNAs in infected cell lines, peripheral blood mononuclear cells (PBMCs), or macrophages.^78^ These conflicting results may be due to different *in vitro* culture conditions and therefore their *in vivo* significance is still to be determined. Nevertheless, a more recent study contained precise and detailed descriptions of a viral miRNA acting as transcriptional regulator^94^ (**Figure 1**). miR-H3 derived from the reverse transcriptase (RT) coding region, where there is relatively high sequence conservation among HIV-1 subtypes, has been shown to regulate HIV-1 replication. The precursor sequence of miR-H3 forms the typical loop structure of an immature miRNA and acts as a substrate for Dicer, allowing generation of the mature miR-H3.^94^ Overexpression of miR-H3 increased viral production, while overexpression of mutated variants of miR-H3 significantly impaired viral replication, suggesting that miR-H3 has a replication-enhancing effect. MiR-H3 appeared to act by increasing both transcription and viral protein expression following interaction with the U3 promoter region, specifically with complementary sequences in the TATA box (about 30 bases upstream from the transcription start site) of the 5′ LTR.^94^ This activation has been demonstrated both in tissue culture models of latency and in primary resting CD4^+^ T cells from HIV-1 infected patients receiving cART, suggesting the basis for a possible new reactivation strategy and purging of HIV-1 from latently infected resting memory cells.^94^ Current pharmacological approaches to viral activation involve pan-T-cell activation, or HDACi and are thereby potentially compromised by off-target effects. In contrast, the use of viral specific activation stimuli, such as miR-H3, may provide a more specific reactivation of latent virus with less off-target effects, if efficient delivery mechanisms can be devised.

## Long noncoding RNA in HIV-1 infected cells

Host derived long noncoding RNA have recently been reported as contributing to virus production in HIV-1 infected cells in two different studies.^95,96^ First, the expression level of NEAT1, a lncRNA, was observed to change upon HIV-1 infection of T-cell lines (Jurkat, MT4); and second, the knockdown of NEAT1 by siRNA was shown to enhance virus production through increased nucleus-to-cytoplasm export of Rev-dependent instability element (INS)-containing HIV-1 mRNAs.^95,96^ Similar observations have been made in other viral infections. Elevated NEAT1 expression has been noted in the mouse central nervous system following infection with either Japanese encephalitis virus or Rabies virus.^97,98^ The expression levels of several other host-derived lncRNAs (*e.g.*, BIC, PANDA) are changed after HIV-1 infection. Interestingly, although these lncRNAs have differential expression only in virus-infected cells, however, characterization of their specific functions remains incomplete.^95,96^

## Impact of asRNA in HIV-1 regulation

More than two decades ago the first putative HIV-1 derived asRNA was published. This species was predicted from an *in silico* analysis of the viral genome and resulted in a prediction that asRNA could encode a protein with an open reading frame (ORF) corresponding to an antisense sequence found within a conserved region of the sense encoded *env* gene.^99^ This prediction was confirmed by identification of a polyadenylated (poly-A) mRNA with negative strand polarity corresponding to this ORF,^100^ which encoded a highly conserved 189-amino-acid polypeptide. However, the function of this protein remains unknown. A novel negative-strand promoter function was also described within the viral 3′-LTR and the activity of this promoter was downregulated by coexpression of Tat. More recent studies have described detection of other asRNAs in HIV-1 infected cells.^19,101,102^ However, a precise role for asRNA in the regulation in the viral life cycle has not been described.

While functional asRNAs have been described in other retroviruses, including HTLV-1 and Maloney murine leukemia virus, the best described role for asRNA in gene regulation in humans is in X-chromosome inactivation. Interestingly, this process is associated with intensive heterochromatin formation mediated by epigenetic mechanisms.^103^ Together these findings suggest that asRNA may potentially be a significant regulator of both host and viral gene expression.

A virus derived asRNA, called ASP-L, has been proposed to influence replication by regulating transcriptional silencing at the HIV-1 promoter.^104^ The predicted promoter of ASP-L is located within the U5 region of the 3′-LTR and includes a putative TATA box, indicating a potential transcriptional start site in an antisense orientation. The data suggested that the long asRNA transcripts interacted with the U3 region of the 5′ LTR to suppress HIV-1 transcription. The expression level of ASP-L was 100–2,500 times less than sense RNA transcripts. Overexpression of ASP-L resulted in significant reductions in viral replication, suggesting a novel mechanism of regulating HIV-transcription and replication.^104^ The innate drivers of expression of this asRNA are not well described outside tissue culture models and the significance of this asRNA's role *in vivo* requires further investigation.

The asRNAs described above are synthesized with polyadenylated modifications. Several studies have found that some antisense ncRNAs lack poly-A tails,^16,20^ suggesting they are retained within the nucleus^16^ (**Figure 1**). The predicted promoter regions of these asRNAs are located in negative strand sequences complementary to the *nef*-coding region with long antisense transcripts extending to the 5′ LTR region, thereby encompassing the majority of the viral genome. So far, these have only been described in various *in vitro* models of HIV-1 latency, including the cell lines ACH2 and J1.1. Interestingly, down modulation of the expression of these long antisense transcripts, achieved by either PTGS or TGS approaches, results in activation of HIV-1 gene expression.^16^ Using a PTGS approach, activation of viral gene expression could be induced by degradation of the long antisense transcript by a small single stranded-antisense RNA (as154) targeting a sequence in the *env* coding region.^16^ Using the TGS approach, a small single stranded RNA complementary to the putative asRNA promoter region was found to induce inhibition of the antisense transcripts by a mechanism consistent with TGS. Both strategies resulted in a reduction of the long antisense transcripts and concurrent activation of sense transcription from the 5′ LTR.^16^ Reduction in expression levels of the long antisense transcript induced lower levels of H3K27me3 and H3K9me2 in chromatin associated with the 5′ LTR in latently infected cell lines and, importantly, activation of latent HIV-1 in an *ex vivo* primary memory CD4^+^ T-cell–based model.^16^ However, the level of induced activation was marginal. Together these data suggest that long antisense transcripts control, or at least impact on, the induction or maintenance of HIV-1 latency. Since the viral activation levels obtained from *ex vivo* primary memory CD4^+^ T cells are not substantial, these interesting findings require further investigation to determine if there are ways of enhancing the potency of this effect. More importantly, since this activation mechanism is sequence specific, it might overcome the current difficulties in eradication strategies, where the current activation strategies result in global T-cell activation.

## siRNA activation of transcription

Similar to the function of miRNAs and asRNAs described above, certain siRNAs are able to induce gene activation. This effect is somewhat target dependent.^105,106,107,108^ The initial reports of siRNA mediated activation related to three genes: E-cadherin, p21, and vascular endothelial growth factor (VEGF) and the effect was shown in prostate cancer cell lines, PC-3 and DU-145, and the human breast cancer cell line, MCF-7.^105^ The authors further investigated the mechanism of transcriptional activation of p21 in the PC-3 cell line and identified the antisense strand of siRNA and Ago2 as being the critical components for this activation, which was associated with loss of methylation of lysine-9 of histone 3 in the chromatin adjacent to dsRNA-target sites. Subsequently, several duplex siRNAs were shown to upregulate transcription of the progesterone receptor in a human breast cancer cell line.^106,109^ This activation was also sequence specific and was accompanied by epigenetic changes, including increased di- and tri-methylation of H3K4.^106^ Other studies have demonstrated that promoter targeted siRNAs can activate transcription of murine VEGF associated with very similar epigenetic changes.^106^

Studies in Drosophila support a role for Ago2 in promoter-associated siRNA transcriptional gene activation,^110,111^ suggesting that this specific protein tends to bind accessible promoter regions where euchromatin predominates, thereby allowing active transcription to take place.^112^ However, more recent studies have revealed that Ago2 is also involved in negative transcriptional regulation and is associated with heterochromatin formation and recruitment of Polycomb group (PcG) transcriptional repressor proteins.^113^ We have also observed involvement of both Ago1 and Ago2 proteins in siRNA mediated TGS of the HIV-1 promoter region,^114^ although the precise role of both proteins is still being investigated (see below section). Therefore, Ago2 does not appear to be a factor in determining whether a siRNA will be inhibitory or activating, when directed at a target within the promoter.

Transcriptional regulatory machinery and their mechanisms are extremely complicated. The critical molecules include: the antisense strand of the siRNA duplex, Ago1 and Ago2, which play different but complementary roles depending on the cell type and finally, the genes and specific region of the gene targeted by the sequence. The precise molecular mechanisms as to whether small RNAs will induce activation or silencing of the promoter are still largely unknown. These findings reveal a diverse role for siRNA molecules in the regulation of gene expression and identify a potential therapeutic use for dsRNA in targeted gene activation and suppression.

## siRNA inhibition of HIV-1 transcription

Transcriptional gene silencing (TGS) induced by siRNA was originally reported in plants and has been reported in certain human cells. Our laboratory was the first to report TGS mediated by a siRNA targeting the HIV-1 promoter. Initial reports were based on *in vitro* work in tissue culture models of HIV-1 infection and showed that four different targets (Prom-A, -B, -C, and -D) mediated substantially different outcomes in terms of viral suppression^115,116,117,118,119,120^ (**Figure 1**). Subsequently, a number of studies have explored the effects of promoter-targeted siRNAs with a variety of targets throughout the HIV-1 promoter region (LTR-247, LTR-336, and LTR-362) (**Figure 1**). The HIV-1 promoter is ~450 bases long and there are several motifs for host transcription factors (*e.g.*, NF-κB, NFAT, SP1, and AP-1) that act to increase HIV-1 transcription. The host transcription repressor, YY1, may also contribute to HIV-1 latency^121^ (**Figure 1**).

The nucleosomes, nuc-0 and nuc-1, are precisely positioned in the HIV-1 LTR after integration of viral DNA into the host genome.^122^ As can be seen from **Figure 1**, a number of sites across the whole HIV-1 promoter region have been used as siRNA target sequences, aimed at induction of TGS. The 5′ LTR is essentially flanked by two nucleosomes, nuc-0, located close to the 5′ end of the LTR, and nuc-1, located in the “R-region.” The region between two nucleosomes is an “open” region containing many of the binding motifs of various host transcription factors. We originally designed HIV-Prom-A around the NF-κB region, as this region contains two NF-κB binding sites arranged in tandem, separated by a four-nucleotide gap, and is essential for HIV-transcription, and highly conserved among different HIV-1 subtypes.^115,116,119^ The nucleotide sequence within the NF-κB binding motif appears to be optimized for HIV-1 gene expression and differs from that found in the promoter regions of human genes which facilitates target specificity.^123^ There are no significant endogenous targets, as a BLAST search of the human genome failed to identify sequences homologous to HIV-Prom-A siRNA.

There are now several studies describing TGS induced by siRNAs targeting NF-κB binding regions. A recent study describes a siRNA named “S4” targeting the NF-κB binding region of HIV-1 subtype-C.^124^ Subtype C has a modified sequence architecture in this region, including three possible NF-κB binding regions, and the sequences these motifs are different from other subtypes.^124^ The S4-siRNA, targeting this critical region of the 5′ LTR, like Prom-A, suppresses virus *in vitro* for more than 2 weeks after a single transfection of the siRNA into human PBMCs. The suppression is accompanied by increased levels of histone methylation, which leads to heterochromatin formation in the targeted U3 region of the 5′ LTR. Interestingly, S4-siRNA is tolerant to certain mismatches in the target region as it suppresses a primary isolate with a one base pair mismatch at position 2 from the 3′ end of the target sequence. The equivalence of this suppression in the presence of these mismatches requires further confirmation. This could be achieved by varying the titers of viral challenge and observing the cultures for longer periods of time. This target is of particular importance since more than half the HIV-1 infections worldwide are caused by subtype-C.^124^

As described in the above section discussing transcriptional regulation mediated through promoter-associated miRNAs, the Argonaute proteins, Ago1 and/or Ago2, form the basis of the RNA-induced transcriptional silencing-like (RITS-like) complex. In this complex, the antisense strand of duplexed siRNA is captured by Ago1 or Ago2. This complex directs siRNA-mediated TGS in mammalian cells and appears to play an important role in both positive and negative regulation of transcriptional activity.^125,126,127^ Our confocal scanning microscopy analysis using fluorescently labeled siPromA siRNA and Flag-tagged Ago1 and Ago2 confirmed that the antisense strand of the siRNA is responsible for induction of TGS.^114^ This analysis also revealed different contributions from both Ago1 and Ago2 in TGS induction: Ago1 colocalized with siRNA in the nucleus, while Ago2 colocalized with siRNA at the inner nuclear envelope of HIV-infected cells. On the other hand, mismatched and scrambled control siRNAs were only observed in the cytoplasm of HIV-infected cells. This was the first report directly visualizing the distribution of Ago-associated siRNA in the nuclear compartment. Our analysis also revealed that the nuclear trafficking mechanism for RITS-like components involved the actin cytoskeleton.^114^ The actin association was consistent when using either SIV and HIV-1 targeted constructs,^128^ suggesting this pathway is not unique to HIV-1.

**Figure 2:**
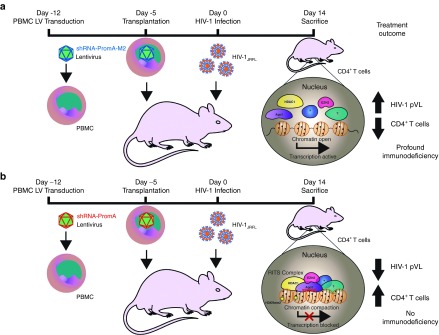
**Graphic representation of the *in vivo* effects of a promoter targeted siRNA approach in a humanized mouse model.** (**a**–**b**) Replication-incompetent lentivirus, (**b**) shPromA-JRFL or (**a**) control lentivirus shPromA-M2, is transfected into CD4^+^ T cells obtained from healthy control PBMCs. Transduced PBMCs are transplanted into (NOD)/SCID/Janus kinase 3 (NOJ) knockout mice, transplantation occurs. The humanized mice are then challenged with HIV-1 and sacrificed 14 days postchallenge. The antisense strand of shPromA-JRFL (shown in red), in association with Ago1 (purple) and other RITS-like complex components (HDAC-yellow, EZH2-pink), induces heterochromatin formation and methylation marks (H3K9me2, indicated by stars) in the targeted HIV-1 promoter region to suppress HIV-1 transcription and results in protection of CD4^+^ T cells, resulting in lower pVL than mice transplanted with shPromA-M2 control lentivirus transduced PBMCs.

We have recently demonstrated the TGS activity of our Prom A construct in an *in vivo* study using the (NOD)/SCID/Janus kinase 3 (NOJ) knockout humanized mouse model (**Figure 2**),^120^ where we employed a lentiviral vector to deliver the short hairpin (sh) form of PromA. After the shRNA expression unit is transcribed from the U6 promoter by RNA polymerase III, which terminates at a poly(T) motif coded within the expression unit, both sense and antisense strands are hybridized with a loop sequence linking both strands. The loop sequence is then processed by cellular ribonucleases to form mature/processed double-stranded siRNA-PromA.^129^ We demonstrated the potential of this approach by reconstituting mice with human PBMC transduced with the lentiviral shPromA construct. This mimics a CD4^+^ T-cell delivery approach. Upon challenge with HIV-1_JRFL_, mice reconstituted with untransduced PBMCs had an acute, progressive HIV-1 infection with high viral loads, massive CD4^+^ T-cell depletion and profound immunodeficiency that occurred within 2 weeks.^130,131^ Despite this acute, rapidly progressive and aggressive infection, pVL in the mice transplanted with PBMC expressing shPromA was significantly lower than in mice treated with the control mutated shPromA-M2 construct, which does not efficiently suppress the virus *in vitro*. Mononuclear cells were recovered at sacrifice (day 14 after HIV-1 infection) from the peritoneal cavity and spleen, and CD4^+^ T cells were markedly reduced relative to CD8^+^ T in the control mice, while the ratio was relatively preserved in mice transplanted with shPromA-transduced cells. Experiments using mice reconstituted with transduced human CD34 cells are currently underway. The aim is to provide sufficient data using this model system to warrant therapeutic development of these constructs. Our proposed therapeutic strategy would be to use these constructs to enforce latency in patients who have been pretreated with cART and have a residual latent viral reservoir. This strategy offers an alternative approach as a “functional cure,” which is the antithesis of a viral activation or “kick and kill” approach. We hypothesize that this new approach could be used in order to induce and maintain HIV-1 latency, enforcing transcriptionally inactive virus even in patients ceasing cART.

## Conclusions

There are an increasing number of studies describing ncRNAs, including asRNA, miRNA, and siRNA, and their ability to modulate HIV-1 transcription, by direct interactions with the HIV-1 U3 promoter region in the 5′ LTR. Mechanisms as to how ncRNAs induce this regulation are still being discovered; however, central players are the Argonaute proteins (Ago1 and Ago2). Argonaute proteins have a PAZ domain that captures the 3′ end of small RNAs to enable the induction of bifunctional effects.^132^ The outcome is dependent on the targeted promoter and probably also on availability and the relative abundance of different species of ncRNAs, including long asRNAs, miRNAs, and antisense-siRNAs in latently infected cells. Although the development of prediction algorithms for the selection of ncRNA sequences against the targeted promoter region has been attempted based on the results of silencing or activation of oncogenes,^133^ further research is required to reveal the exact mechanisms that induce up- or downregulation of transcription.

asRNA, miRNA, and siRNAs have all been shown to interact with targets distributed across the entirety of the HIV-1 U3 promoter region and regulate transcription, both positively and negatively. The biggest potential advantage of ncRNA meditated transcriptional regulation of HIV-1, particularly in the context of latently infected cells, is their exquisite sequence specificity, which enables a more targeted approach to manipulation of the reservoir. This is in distinct contrast to the current activation approach, which depends on general T-cell activation or widespread alterations in histone acetylation that can result in off-target effects. In the future, ncRNA-based approaches may lead to novel therapies that either enforce HIV-1 latent infection or induce activation of HIV from latently infected cells, allowing control of the reservoir in the absence of traditional antiretroviral drugs.

## References

[bib1] Birney E Stamatoyannopoulos JA Dutta A Guigó R Gingeras TR Margulies EH*et al*. (2007 Identification and analysis of functional elements in 1% of the human genome by the ENCODE pilot project Nature 447 799 816 17571346 10.1038/nature05874 PMC2212820

[bib2] Kaikkonen MU Lam MT Glass CK 2011 Non-coding RNAs as regulators of gene expression and epigenetics Cardiovasc Res 90 430 440 21558279 10.1093/cvr/cvr097 PMC3096308

[bib3] Malecová B Morris KV 2010 Transcriptional gene silencing through epigenetic changes mediated by non-coding RNAs Curr Opin Mol Ther 12 214 222 20373265 PMC2861437

[bib4] Morris KV Mattick JS 2014 The rise of regulatory RNA Nat Rev Genet 15 423 437 24776770 10.1038/nrg3722 PMC4314111

[bib5] Stefani G Slack FJ 2008 Small non-coding RNAs in animal development Nat Rev Mol Cell Biol 9 219 230 18270516 10.1038/nrm2347

[bib6] Gibb EA Brown CJ Lam WL 2011 The functional role of long non-coding RNA in human carcinomas Mol Cancer 10 38 21489289 10.1186/1476-4598-10-38 PMC3098824

[bib7] Weinberg MS Morris KV 2013 Long non-coding RNA targeting and transcriptional de-repression Nucleic Acid Ther 23 9 14 23391414 10.1089/nat.2012.0412 PMC3569965

[bib8] Morris KV 2012 *Non-Coding RNAs and Epigenetic Regulation of Gene Expression* Caister Acadimic PressNorfolk

[bib9] Prensner JR Chinnaiyan AM 2011 The emergence of lncRNAs in cancer biology Cancer Discov 1 391 407 22096659 10.1158/2159-8290.CD-11-0209 PMC3215093

[bib10] Siliciano RF Greene WC 2011 HIV latency Cold Spring Harb Perspect Med 1 a007096 22229121 10.1101/cshperspect.a007096 PMC3234450

[bib11] Schiralli Lester GM Henderson AJ 2012 Mechanisms of HIV Transcriptional Regulation and Their Contribution to Latency Mol Biol Int 2012 614120 22701796 10.1155/2012/614120 PMC3371693

[bib12] Kent SJ Reece JC Petravic J Martyushev A Kramski M De Rose R*et al*. (2013 The search for an HIV cure: tackling latent infection Lancet Infect Dis 13 614 621 23481675 10.1016/S1473-3099(13)70043-4

[bib13] Ho YC Shan L Hosmane NN Wang J Laskey SB Rosenbloom DI*et al*. (2013 Replication-competent noninduced proviruses in the latent reservoir increase barrier to HIV-1 cure Cell 155 540 551 24243014 10.1016/j.cell.2013.09.020 PMC3896327

[bib14] Koiwa T Hamano-Usami A Ishida T Okayama A Yamaguchi K Kamihira S*et al*. (2002 5'-long terminal repeat-selective CpG methylation of latent human T-cell leukemia virus type 1 provirus *in vitro* and in vivo J Virol 76 9389 9397 12186921 10.1128/JVI.76.18.9389-9397.2002 PMC136445

[bib15] Brooks DG Arlen PA Gao L Kitchen CM Zack JA 2003 Identification of T cell-signaling pathways that stimulate latent HIV in primary cells Proc Natl Acad Sci USA 100 12955 12960 14569007 10.1073/pnas.2233345100 PMC240726

[bib16] Saayman S Ackley A Turner AM Famiglietti M Bosque A Clemson M*et al*. (2014 An HIV-encoded antisense long noncoding RNA epigenetically regulates viral transcription Mol Ther 22 1164 1175 24576854 10.1038/mt.2014.29 PMC4048891

[bib17] Kurokawa R 2011 Promoter-associated long noncoding RNAs repress transcription through a RNA binding protein TLS Adv Exp Med Biol 722 196 208 21915790 10.1007/978-1-4614-0332-6_12

[bib18] Kurokawa R Rosenfeld MG Glass CK 2009 Transcriptional regulation through noncoding RNAs and epigenetic modifications RNA Biol 6 233 236 19411842 10.4161/rna.6.3.8329

[bib19] Schopman NC Willemsen M Liu YP Bradley T van Kampen A Baas F*et al*. (2012 Deep sequencing of virus-infected cells reveals HIV-encoded small RNAs Nucleic Acids Res 40 414 427 21911362 10.1093/nar/gkr719 PMC3245934

[bib20] Eilebrecht S Schwartz C Rohr O 2013 Non-coding RNAs: novel players in chromatin-regulation during viral latency Curr Opin Virol 3 387 393 23660570 10.1016/j.coviro.2013.04.001

[bib21] Ho DD Neumann AU Perelson AS Chen W Leonard JM Markowitz M 1995 Rapid turnover of plasma virions and CD4 lymphocytes in HIV-1 infection Nature 373 123 126 7816094 10.1038/373123a0

[bib22] Dinoso JB Kim SY Wiegand AM Palmer SE Gange SJ Cranmer L*et al*. (2009 Treatment intensification does not reduce residual HIV-1 viremia in patients on highly active antiretroviral therapy Proc Natl Acad Sci USA 106 9403 9408 19470482 10.1073/pnas.0903107106 PMC2685743

[bib23] Wong JK Hezareh M Günthard HF Havlir DV Ignacio CC Spina CA*et al*. (1997 Recovery of replication-competent HIV despite prolonged suppression of plasma viremia Science 278 1291 1295 9360926 10.1126/science.278.5341.1291

[bib24] Chun TW Justement JS Moir S Hallahan CW Maenza J Mullins JI*et al*. (2007 Decay of the HIV reservoir in patients receiving antiretroviral therapy for extended periods: implications for eradication of virus J Infect Dis 195 1762 1764 17492591 10.1086/518250

[bib25] Mellors JW Rinaldo CR Jr Gupta P White RM Todd JA Kingsley LA 1996 Prognosis in HIV-1 infection predicted by the quantity of virus in plasma Science 272 1167 1170 8638160 10.1126/science.272.5265.1167

[bib26] Perelson AS Neumann AU Markowitz M Leonard JM Ho DD 1996 HIV-1 dynamics in vivo: virion clearance rate, infected cell life-span, and viral generation time Science 271 1582 1586 8599114 10.1126/science.271.5255.1582

[bib27] Gulick RM Mellors JW Havlir D Eron JJ Gonzalez C McMahon D*et al*. (1997 Treatment with indinavir, zidovudine, and lamivudine in adults with human immunodeficiency virus infection and prior antiretroviral therapy N Engl J Med 337 734 739 9287228 10.1056/NEJM199709113371102

[bib28] Palella FJ Jr Delaney KM Moorman AC Loveless MO Fuhrer J Satten GA*et al*. (1998 Declining morbidity and mortality among patients with advanced human immunodeficiency virus infection. HIV Outpatient Study Investigators N Engl J Med 338 853 860 9516219 10.1056/NEJM199803263381301

[bib29] Chun TW Finzi D Margolick J Chadwick K Schwartz D Siliciano RF 1995 *In vivo* fate of HIV-1-infected T cells: quantitative analysis of the transition to stable latency Nat Med 1 1284 1290 7489410 10.1038/nm1295-1284

[bib30] Chun TW Carruth L Finzi D Shen X DiGiuseppe JA Taylor H*et al*. (1997 Quantification of latent tissue reservoirs and total body viral load in HIV-1 infection Nature 387 183 188 9144289 10.1038/387183a0

[bib31] Finzi D Hermankova M Pierson T Carruth LM Buck C Chaisson RE*et al*. (1997 Identification of a reservoir for HIV-1 in patients on highly active antiretroviral therapy Science 278 1295 1300 9360927 10.1126/science.278.5341.1295

[bib32] Burnett JC Lim KI Calafi A Rossi JJ Schaffer DV Arkin AP 2010 Combinatorial latency reactivation for HIV-1 subtypes and variants J Virol 84 5958 5974 20357084 10.1128/JVI.00161-10 PMC2876650

[bib33] Mohammadi P di Iulio J Muñoz M Martinez R Bartha I Cavassini M*et al*. (2014 Dynamics of HIV latency and reactivation in a primary CD4+ T cell model PLoS Pathog 10 e1004156 24875931 10.1371/journal.ppat.1004156 PMC4038609

[bib34] van Praag RM Prins JM Roos MT Schellekens PT Ten Berge IJ Yong SL*et al*. (2001 OKT3 and IL-2 treatment for purging of the latent HIV-1 reservoir *in vivo* results in selective long-lasting CD4+ T cell depletion J Clin Immunol 21 218 226 11403229 10.1023/a:1011091300321

[bib35] Paiardini M 2011 Editorial: Hijacking the IL-7/IL-7R system in HIV infection J Leukoc Biol 89 491 493 21454359 10.1189/jlb.1110614

[bib36] Wang FX Xu Y Sullivan J Souder E Argyris EG Acheampong EA*et al*. (2005 IL-7 is a potent and proviral strain-specific inducer of latent HIV-1 cellular reservoirs of infected individuals on virally suppressive HAART J Clin Invest 115 128 137 15630452 10.1172/JCI22574 PMC539197

[bib37] McKernan LN Momjian D Kulkosky J 2012 Protein Kinase C: One Pathway towards the Eradication of Latent HIV-1 Reservoirs Adv Virol 2012 805347 22500169 10.1155/2012/805347 PMC3303757

[bib38] Weissman D Dybul M Daucher MB Davey RT Jr Walker RE Kovacs JA 2000 Interleukin-2 up-regulates expression of the human immunodeficiency virus fusion coreceptor CCR5 by CD4+ lymphocytes in vivo J Infect Dis 181 933 938 10720515 10.1086/315303

[bib39] Sánchez-Duffhues G Vo MQ Pérez M Calzado MA Moreno S Appendino G*et al*. (2011 Activation of latent HIV-1 expression by protein kinase C agonists. A novel therapeutic approach to eradicate HIV-1 reservoirs Curr Drug Targets 12 348 356 20955147 10.2174/138945011794815266

[bib40] Duvic M Talpur R Ni X Zhang C Hazarika P Kelly C*et al*. (2007 Phase 2 trial of oral vorinostat (suberoylanilide hydroxamic acid, SAHA) for refractory cutaneous T-cell lymphoma (CTCL) Blood 109 31 39 16960145 10.1182/blood-2006-06-025999 PMC1785068

[bib41] Edelstein LC Micheva-Viteva S Phelan BD Dougherty JP 2009 Short communication: activation of latent HIV type 1 gene expression by suberoylanilide hydroxamic acid (SAHA), an HDAC inhibitor approved for use to treat cutaneous T cell lymphoma AIDS Res Hum Retroviruses 25 883 887 19689202 10.1089/aid.2008.0294 PMC2828260

[bib42] Heider U Rademacher J Lamottke B Mieth M Moebs M von Metzler I*et al*. (2009 Synergistic interaction of the histone deacetylase inhibitor SAHA with the proteasome inhibitor bortezomib in cutaneous T cell lymphoma Eur J Haematol 82 440 449 19220424 10.1111/j.1600-0609.2009.01239.x

[bib43] Wei DG Chiang V Fyne E Balakrishnan M Barnes T Graupe M*et al*. (2014 Histone deacetylase inhibitor romidepsin induces HIV expression in CD4 T cells from patients on suppressive antiretroviral therapy at concentrations achieved by clinical dosing PLoS Pathog 10 e1004071 24722454 10.1371/journal.ppat.1004071 PMC3983056

[bib44] Battistini A Sgarbanti M 2014 HIV-1 latency: an update of molecular mechanisms and therapeutic strategies Viruses 6 1715 1758 24736215 10.3390/v6041715 PMC4014718

[bib45] Jones RB O'Connor R Mueller S Foley M Szeto GL Karel D*et al*. (2014 Histone deacetylase inhibitors impair the elimination of HIV-infected cells by cytotoxic T-lymphocytes PLoS Pathog 10 e1004287 25122219 10.1371/journal.ppat.1004287 PMC4133386

[bib46] Sampey GC Guendel I Das R Jaworski E Klase Z Narayanan A*et al*. (2012 Transcriptional Gene Silencing (TGS) via the RNAi Machinery in HIV-1 Infections Biology (Basel) 1 339 369 24832229 10.3390/biology1020339 PMC4009781

[bib47] Groen JN Morris KV 2013 Chromatin, non-coding RNAs, and the expression of HIV Viruses 5 1633 1645 23812489 10.3390/v5071633 PMC3738951

[bib48] Lee RC Feinbaum RL Ambros V 1993 The C. elegans heterochronic gene lin-4 encodes small RNAs with antisense complementarity to lin-14 Cell 75 843 854 8252621 10.1016/0092-8674(93)90529-y

[bib49] Kim VN Han J Siomi MC 2009 Biogenesis of small RNAs in animals Nat Rev Mol Cell Biol 10 126 139 19165215 10.1038/nrm2632

[bib50] Fu J Tang W Du P Wang G Chen W Li J*et al*. (2012 Identifying microRNA-mRNA regulatory network in colorectal cancer by a combination of expression profile and bioinformatics analysis BMC Syst Biol 6 68 22703586 10.1186/1752-0509-6-68 PMC3418553

[bib51] Grey F Tirabassi R Meyers H Wu G McWeeney S Hook L*et al*. (2010 A viral microRNA down-regulates multiple cell cycle genes through mRNA 5'UTRs PLoS Pathog 6 e1000967 20585629 10.1371/journal.ppat.1000967 PMC2891821

[bib52] Kim DH Saetrom P Snøve O Jr Rossi JJ 2008 MicroRNA-directed transcriptional gene silencing in mammalian cells Proc Natl Acad Sci USA 105 16230 16235 18852463 10.1073/pnas.0808830105 PMC2571020

[bib53] Younger ST Corey DR 2011 Transcriptional gene silencing in mammalian cells by miRNA mimics that target gene promoters Nucleic Acids Res 39 5682 5691 21427083 10.1093/nar/gkr155 PMC3141263

[bib54] Place RF Li LC Pookot D Noonan EJ Dahiya R 2008 MicroRNA-373 induces expression of genes with complementary promoter sequences Proc Natl Acad Sci USA 105 1608 1613 18227514 10.1073/pnas.0707594105 PMC2234192

[bib55] Majid S Dar AA Saini S Yamamura S Hirata H Tanaka Y*et al*. (2010 MicroRNA-205-directed transcriptional activation of tumor suppressor genes in prostate cancer Cancer 116 5637 5649 20737563 10.1002/cncr.25488 PMC3940365

[bib56] Huang J Wang F Argyris E Chen K Liang Z Tian H*et al*. (2007 Cellular microRNAs contribute to HIV-1 latency in resting primary CD4+ T lymphocytes Nat Med 13 1241 1247 17906637 10.1038/nm1639

[bib57] Wang X Ye L Hou W Zhou Y Wang YJ Metzger DS*et al*. (2009 Cellular microRNA expression correlates with susceptibility of monocytes/macrophages to HIV-1 infection Blood 113 671 674 19015395 10.1182/blood-2008-09-175000 PMC2628373

[bib58] Cobos Jiménez V Booiman T de Taeye SW van Dort KA Rits MA Hamann J*et al*. (2012 Differential expression of HIV-1 interfering factors in monocyte-derived macrophages stimulated with polarizing cytokines or interferons Sci Rep 2 763 23094138 10.1038/srep00763 PMC3478582

[bib59] Mantri CK Pandhare Dash J Mantri JV Dash CC 2012 Cocaine enhances HIV-1 replication in CD4+ T cells by down-regulating MiR-125b PLoS ONE 7 e51387 23251514 10.1371/journal.pone.0051387 PMC3520918

[bib60] Wang X Ye L Zhou Y Liu MQ Zhou DJ Ho WZ 2011 Inhibition of anti-HIV microRNA expression: a mechanism for opioid-mediated enhancement of HIV infection of monocytes Am J Pathol 178 41 47 21224041 10.1016/j.ajpath.2010.11.042 PMC3069926

[bib61] Swaminathan S Zaunders J Wilkinson J Suzuki K Kelleher AD 2009 Does the presence of anti-HIV miRNAs in monocytes explain their resistance to HIV-1 infection Blood 113 5029 30; author reply 5030 19443673 10.1182/blood-2009-01-196741

[bib62] Sun G Li H Wu X Covarrubias M Scherer L Meinking K*et al*. (2012 Interplay between HIV-1 infection and host microRNAs Nucleic Acids Res 40 2181 2196 22080513 10.1093/nar/gkr961 PMC3300021

[bib63] Betel D Wilson M Gabow A Marks DS Sander C 2008 The microRNA.org resource: targets and expression Nucleic Acids Res 36 Database issue D149 D153 18158296 10.1093/nar/gkm995 PMC2238905

[bib64] Landgraf P Rusu M Sheridan R Sewer A Iovino N Aravin A*et al*. (2007 A mammalian microRNA expression atlas based on small RNA library sequencing Cell 129 1401 1414 17604727 10.1016/j.cell.2007.04.040 PMC2681231

[bib65] Swaminathan G Navas-Martín S Martín-García J 2014 Interplay between microRNAs, Toll-like receptors, and HIV-1: potential implications in HIV-1 replication and chronic immune activation Discov Med 18 15 27 25091485

[bib66] Swaminathan S Kelleher AD 2014 MicroRNA modulation of key targets associated with T cell exhaustion in HIV-1 infection Curr Opin HIV AIDS 9 464 471 25023625 10.1097/COH.0000000000000089

[bib67] Zhang HS Chen XY Wu TC Sang WW Ruan Z 2012 MiR-34a is involved in Tat-induced HIV-1 long terminal repeat (LTR) transactivation through the SIRT1/NF?B pathway FEBS Lett 586 4203 4207 23103739 10.1016/j.febslet.2012.10.023

[bib68] Zhang HS Wu TC Sang WW Ruan Z 2012 MiR-217 is involved in Tat-induced HIV-1 long terminal repeat (LTR) transactivation by down-regulation of SIRT1 Biochim Biophys Acta 1823 1017 1023 22406815 10.1016/j.bbamcr.2012.02.014

[bib69] Chen XY Zhang HS Wu TC Sang WW Ruan Z 2013 Down-regulation of NAMPT expression by miR-182 is involved in Tat-induced HIV-1 long terminal repeat (LTR) transactivation Int J Biochem Cell Biol 45 292 298 23153509 10.1016/j.biocel.2012.11.002

[bib70] Shen CJ Jia YH Tian RR Ding M Zhang C Wang JH 2012 Translation of Pur-a is targeted by cellular miRNAs to modulate the differentiation-dependent susceptibility of monocytes to HIV-1 infection FASEB J 26 4755 4764 22835829 10.1096/fj.12-209023

[bib71] Ma L Shen CJ Cohen EA Xiong SD Wang JH 2014 miRNA-1236 inhibits HIV-1 infection of monocytes by repressing translation of cellular factor VprBP PLoS ONE 9 e99535 24932481 10.1371/journal.pone.0099535 PMC4059663

[bib72] Triboulet R Mari B Lin YL Chable-Bessia C Bennasser Y Lebrigand K*et al*. (2007 Suppression of microRNA-silencing pathway by HIV-1 during virus replication Science 315 1579 1582 17322031 10.1126/science.1136319

[bib73] Swaminathan S Murray DD Kelleher AD 2013 miRNAs and HIV: unforeseen determinants of host-pathogen interaction Immunol Rev 254 265 280 23772625 10.1111/imr.12077

[bib74] Zhou Y Wang X Liu M Hu Q Song L Ye L*et al*. (2010 A critical function of toll-like receptor-3 in the induction of anti-human immunodeficiency virus activities in macrophages Immunology 131 40 49 20636339 10.1111/j.1365-2567.2010.03270.x PMC2966756

[bib75] Swaminathan G Rossi F Sierra LJ Gupta A Navas-Martín S Martín-García J 2012 A role for microRNA-155 modulation in the anti-HIV-1 effects of Toll-like receptor 3 stimulation in macrophages PLoS Pathog 8 e1002937 23028330 10.1371/journal.ppat.1002937 PMC3447756

[bib76] Hicks J Liu HC 2013 Involvement of eukaryotic small RNA pathways in host defense and viral pathogenesis Viruses 5 2659 2678 24178713 10.3390/v5112659 PMC3856408

[bib77] Duskova K Nagilla P Le HS Iyer P Thalamuthu A Martinson J*et al*. (2013 MicroRNA regulation and its effects on cellular transcriptome in human immunodeficiency virus-1 (HIV-1) infected individuals with distinct viral load and CD4 cell counts BMC Infect Dis 13 250 23721325 10.1186/1471-2334-13-250 PMC3680326

[bib78] Whisnant AW Bogerd HP Flores O Ho P Powers JG Sharova N*et al*. (2013 In-depth analysis of the interaction of HIV-1 with cellular microRNA biogenesis and effector mechanisms MBio 4 e000193 23592263 10.1128/mBio.00193-13 PMC3634607

[bib79] Donahue DA Wainberg MA 2013 Cellular and molecular mechanisms involved in the establishment of HIV-1 latency Retrovirology 10 11 23375003 10.1186/1742-4690-10-11 PMC3571915

[bib80] Noguchi K Ishibashi K Miyokawa K Hokari M Kanno T Hirano T*et al*. (2012 HIV-1 suppressive sequences are modulated by Rev transport of unspliced RNA and are required for efficient HIV-1 production PLoS ONE 7 e51393 23251516 10.1371/journal.pone.0051393 PMC3519575

[bib81] Chiang K Rice AP 2012 MicroRNA-mediated restriction of HIV-1 in resting CD4+ T cells and monocytes Viruses 4 1390 1409 23170164 10.3390/v4091390 PMC3499811

[bib82] Chiang K Liu H Rice AP 2013 miR-132 enhances HIV-1 replication Virology 438 1 4 23357732 10.1016/j.virol.2012.12.016 PMC3594373

[bib83] Klase Z Houzet L Jeang KT 2012 MicroRNAs and HIV-1: complex interactions J Biol Chem 287 40884 40890 23043098 10.1074/jbc.R112.415448 PMC3510792

[bib84] Tan Gana NH Onuki T Victoriano AF Okamoto T 2012 MicroRNAs in HIV-1 infection: an integration of viral and cellular interaction at the genomic level Front Microbiol 3 306 22936931 10.3389/fmicb.2012.00306 PMC3426883

[bib85] Omoto S Ito M Tsutsumi Y Ichikawa Y Okuyama H Brisibe EA*et al*. (2004 HIV-1 nef suppression by virally encoded microRNA Retrovirology 1 44 15601474 10.1186/1742-4690-1-44 PMC544868

[bib86] Omoto S Fujii YR 2005 Regulation of human immunodeficiency virus 1 transcription by nef microRNA J Gen Virol 86 Pt 3 751 755 15722536 10.1099/vir.0.80449-0

[bib87] Klase Z Kale P Winograd R Gupta MV Heydarian M Berro R*et al*. (2007 HIV-1 TAR element is processed by Dicer to yield a viral micro-RNA involved in chromatin remodeling of the viral LTR BMC Mol Biol 8 63 17663774 10.1186/1471-2199-8-63 PMC1955452

[bib88] Ouellet DL Plante I Landry P Barat C Janelle ME Flamand L*et al*. (2008 Identification of functional microRNAs released through asymmetrical processing of HIV-1 TAR element Nucleic Acids Res 36 2353 2365 18299284 10.1093/nar/gkn076 PMC2367715

[bib89] Klase Z Winograd R Davis J Carpio L Hildreth R Heydarian M*et al*. (2009 HIV-1 TAR miRNA protects against apoptosis by altering cellular gene expression Retrovirology 6 18 19220914 10.1186/1742-4690-6-18 PMC2654423

[bib90] Althaus CF Vongrad V Niederöst B Joos B Di Giallonardo F Rieder P*et al*. (2012 Tailored enrichment strategy detects low abundant small noncoding RNAs in HIV-1 infected cells Retrovirology 9 27 22458358 10.1186/1742-4690-9-27 PMC3341194

[bib91] Pfeffer S Sewer A Lagos-Quintana M Sheridan R Sander C Grässer FA*et al*. (2005 Identification of microRNAs of the herpesvirus family Nat Methods 2 269 276 15782219 10.1038/nmeth746

[bib92] Lin J Cullen BR 2007 Analysis of the interaction of primate retroviruses with the human RNA interference machinery J Virol 81 12218 12226 17855543 10.1128/JVI.01390-07 PMC2169020

[bib93] Ghosh Z Mallick B Chakrabarti J 2009 Cellular versus viral microRNAs in host-virus interaction Nucleic Acids Res 37 1035 1048 19095692 10.1093/nar/gkn1004 PMC2651794

[bib94] Zhang Y Fan M Geng G Liu B Huang Z Luo H*et al*. (2014 A novel HIV-1-encoded microRNA enhances its viral replication by targeting the TATA box region Retrovirology 11 23 24620741 10.1186/1742-4690-11-23 PMC4007588

[bib95] Zhang Q Chen CY Yedavalli VS Jeang KT 2013 NEAT1 long noncoding RNA and paraspeckle bodies modulate HIV-1 posttranscriptional expression MBio 4 e00596 e00512 23362321 10.1128/mBio.00596-12 PMC3560530

[bib96] Zhang Q Jeang KT 2013 Long non-coding RNAs (lncRNAs) and viral infections Biomed Pharmacother 3 34 42 23645970 10.1016/j.biomed.2013.01.001 PMC3641704

[bib97] Saha S Rangarajan PN 2003 Common host genes are activated in mouse brain by Japanese encephalitis and rabies viruses J Gen Virol 84 Pt 7 1729 1735 12810866 10.1099/vir.0.18826-0

[bib98] Saha S Murthy S Rangarajan PN 2006 Identification and characterization of a virus-inducible non-coding RNA in mouse brain J Gen Virol 87 Pt 7 1991 1995 16760401 10.1099/vir.0.81768-0

[bib99] Miller RH 1988 Human immunodeficiency virus may encode a novel protein on the genomic DNA plus strand Science 239 1420 1422 3347840 10.1126/science.3347840

[bib100] Michael NL Vahey MT d'Arcy L Ehrenberg PK Mosca JD Rappaport J*et al*. (1994 Negative-strand RNA transcripts are produced in human immunodeficiency virus type 1-infected cells and patients by a novel promoter downregulated by Tat J Virol 68 979 987 8289399 10.1128/jvi.68.2.979-987.1994 PMC236536

[bib101] Landry S Halin M Lefort S Audet B Vaquero C Mesnard JM*et al*. (2007 Detection, characterization and regulation of antisense transcripts in HIV-1 Retrovirology 4 71 17910760 10.1186/1742-4690-4-71 PMC2099442

[bib102] Ludwig LB Ambrus JL Jr Krawczyk KA Sharma S Brooks S Hsiao CB*et al*. (2006 Human Immunodeficiency Virus-Type 1 LTR DNA contains an intrinsic gene producing antisense RNA and protein products Retrovirology 3 80 17090330 10.1186/1742-4690-3-80 PMC1654176

[bib103] Lee JT 2009 Lessons from X-chromosome inactivation: long ncRNA as guides and tethers to the epigenome Genes Dev 23 1831 1842 19684108 10.1101/gad.1811209 PMC2725936

[bib104] Kobayashi-Ishihara M Yamagishi M Hara T Matsuda Y Takahashi R Miyake A*et al*. (2012 HIV-1-encoded antisense RNA suppresses viral replication for a prolonged period Retrovirology 9 38 22569184 10.1186/1742-4690-9-38 PMC3410806

[bib105] Li LC Okino ST Zhao H Pookot D Place RF Urakami S*et al*. (2006 Small dsRNAs induce transcriptional activation in human cells Proc Natl Acad Sci USA 103 17337 17342 17085592 10.1073/pnas.0607015103 PMC1859931

[bib106] Janowski BA Younger ST Hardy DB Ram R Huffman KE Corey DR 2007 Activating gene expression in mammalian cells with promoter-targeted duplex RNAs Nat Chem Biol 3 166 173 17259978 10.1038/nchembio860

[bib107] Yang K Shen J Xie YQ Lin YW Qin J Mao QQ*et al*. (2013 Promoter-targeted double-stranded small RNAs activate PAWR gene expression in human cancer cells Int J Biochem Cell Biol 45 1338 1346 23583662 10.1016/j.biocel.2013.03.022

[bib108] Qin Q Lin YW Zheng XY Chen H Mao QQ Yang K*et al*. (2012 RNAa-mediated overexpression of WT1 induces apoptosis in HepG2 cells World J Surg Oncol 10 11 22244202 10.1186/1477-7819-10-11 PMC3268108

[bib109] Turunen MP Lehtola T Heinonen SE Assefa GS Korpisalo P Girnary R*et al*. (2009 Efficient regulation of VEGF expression by promoter-targeted lentiviral shRNAs based on epigenetic mechanism: a novel example of epigenetherapy Circ Res 105 604 609 19696410 10.1161/CIRCRESAHA.109.200774

[bib110] Moshkovich N Nisha P Boyle PJ Thompson BA Dale RK Lei EP 2011 RNAi-independent role for Argonaute2 in CTCF/CP190 chromatin insulator function Genes Dev 25 1686 1701 21852534 10.1101/gad.16651211 PMC3165934

[bib111] Cernilogar FM Onorati MC Kothe GO Burroughs AM Parsi KM Breiling A*et al*. (2011 Chromatin-associated RNA interference components contribute to transcriptional regulation in Drosophila Nature 480 391 395 22056986 10.1038/nature10492 PMC4082306

[bib112] Li LC 2014 Chromatin remodeling by the small RNA machinery in mammalian cells Epigenetics 9 45 52 24149777 10.4161/epi.26830 PMC3928185

[bib113] Taliaferro JM Aspden JL Bradley T Marwha D Blanchette M Rio DC 2013 Two new and distinct roles for Drosophila Argonaute-2 in the nucleus: alternative pre-mRNA splicing and transcriptional repression Genes Dev 27 378 389 23392611 10.1101/gad.210708.112 PMC3589555

[bib114] Ahlenstiel CL Lim HG Cooper DA Ishida T Kelleher AD Suzuki K 2012 Direct evidence of nuclear Argonaute distribution during transcriptional silencing links the actin cytoskeleton to nuclear RNAi machinery in human cells Nucleic Acids Res 40 1579 1595 22064859 10.1093/nar/gkr891 PMC3287199

[bib115] Suzuki K Shijuuku T Fukamachi T Zaunders J Guillemin G Cooper D*et al*. (2005 Prolonged transcriptional silencing and CpG methylation induced by siRNAs targeted to the HIV-1 promoter region J RNAi Gene Silencing 1 66 78 19771207 PMC2737205

[bib116] Suzuki K Juelich T Lim H Ishida T Watanebe T Cooper DA*et al*. (2008 Closed chromatin architecture is induced by an RNA duplex targeting the HIV-1 promoter region J Biol Chem 283 23353 23363 18519571 10.1074/jbc.M709651200 PMC2516975

[bib117] Suzuki K Kelleher AD 2009 Transcriptional regulation by promoter targeted RNAs Curr Top Med Chem 9 1079 1087 19860708 10.2174/156802609789630875

[bib118] Yamagishi M Ishida T Miyake A Cooper DA Kelleher AD Suzuki K*et al*. (2009 Retroviral delivery of promoter-targeted shRNA induces long-term silencing of HIV-1 transcription Microbes Infect 11 500 508 19233310 10.1016/j.micinf.2009.02.003

[bib119] Suzuki K Ishida T Yamagishi M Ahlenstiel C Swaminathan S Marks K*et al*. (2011 Transcriptional gene silencing of HIV-1 through promoter targeted RNA is highly specific RNA Biol 8 1035 1046 21955498 10.4161/rna.8.6.16264 PMC3256422

[bib120] Suzuki K Hattori S Marks K Ahlenstiel C Maeda Y Ishida T*et al*. (2013 Promoter Targeting shRNA Suppresses HIV-1 Infection *In vivo* Through Transcriptional Gene Silencing Mol Ther Nucleic Acids 2 e137 24301868 10.1038/mtna.2013.64 PMC3894581

[bib121] Van Lint C Bouchat S Marcello A 2013 HIV-1 transcription and latency: an update Retrovirology 10 67 23803414 10.1186/1742-4690-10-67 PMC3699421

[bib122] Verdin E Paras P Jr Van Lint C 1993 Chromatin disruption in the promoter of human immunodeficiency virus type 1 during transcriptional activation EMBO J 12 3249 3259 8344262 10.1002/j.1460-2075.1993.tb05994.x PMC413592

[bib123] Chen-Park FE Huang DB Noro B Thanos D Ghosh G 2002 The kappa B DNA sequence from the HIV long terminal repeat functions as an allosteric regulator of HIV transcription J Biol Chem 277 24701 24708 11970949 10.1074/jbc.M200007200

[bib124] Singh A Palanichamy JK Ramalingam P Kassab MA Bhagat M Andrabi R*et al*. (2014 Long-term suppression of HIV-1C virus production in human peripheral blood mononuclear cells by LTR heterochromatization with a short double-stranded RNA J Antimicrob Chemother 69 404 415 24022068 10.1093/jac/dkt348

[bib125] Kim DH Villeneuve LM Morris KV Rossi JJ 2006 Argonaute-1 directs siRNA-mediated transcriptional gene silencing in human cells Nat Struct Mol Biol 13 793 797 16936726 10.1038/nsmb1142

[bib126] Morris KV Chan SW Jacobsen SE Looney DJ 2004 Small interfering RNA-induced transcriptional gene silencing in human cells Science 305 1289 1292 15297624 10.1126/science.1101372

[bib127] Robb GB Brown KM Khurana J Rana TM 2005 Specific and potent RNAi in the nucleus of human cells Nat Struct Mol Biol 12 133 137 15643423 10.1038/nsmb886

[bib128] Lim HG Suzuki K Cooper DA Kelleher AD 2008 Promoter-targeted siRNAs induce gene silencing of simian immunodeficiency virus (SIV) infection *in vitro* Mol Ther 16 565 570 18227841 10.1038/sj.mt.6300380

[bib129] Brummelkamp TR Bernards R Agami R 2002 A system for stable expression of short interfering RNAs in mammalian cells Science 296 550 553 11910072 10.1126/science.1068999

[bib130] Okada S Harada H Ito T Saito T Suzu S 2008 Early development of human hematopoietic and acquired immune systems in new born NOD/Scid/Jak3null mice intrahepatic engrafted with cord blood-derived CD34 + cells Int J Hematol 88 476 482 19039627 10.1007/s12185-008-0215-z

[bib131] Hattori S Ide K Nakata H Harada H Suzu S Ashida N*et al*. (2009 Potent activity of a nucleoside reverse transcriptase inhibitor, 4'-ethynyl-2-fluoro-2'-deoxyadenosine, against human immunodeficiency virus type 1 infection in a model using human peripheral blood mononuclear cell-transplanted NOD/SCID Janus kinase 3 knockout mice Antimicrob Agents Chemother 53 3887 3893 19546363 10.1128/AAC.00270-09 PMC2737856

[bib132] Gu S Jin L Huang Y Zhang F Kay MA 2012 Slicing-independent RISC activation requires the argonaute PAZ domain Curr Biol 22 1536 1542 22795694 10.1016/j.cub.2012.06.040 PMC3604743

[bib133] Ackley A Lenox A Stapleton K Knowling S Lu T Sabir KS*et al*. (2013 An Algorithm for Generating Small RNAs Capable of Epigenetically Modulating Transcriptional Gene Silencing and Activation in Human Cells Mol Ther Nucleic Acids 2 e104 23839098 10.1038/mtna.2013.33 PMC3731886

